# SARS-CoV-2 spike L452R mutation increases Omicron variant fusogenicity and infectivity as well as host glycolysis

**DOI:** 10.1038/s41392-022-00941-z

**Published:** 2022-03-09

**Authors:** Yanan Zhang, Ting Zhang, Yihui Fang, Jie Liu, Qinong Ye, Lihua Ding

**Affiliations:** 1 Department of Medical Molecular Biology, Beijing Institute of Biotechnology, Beijing, 100850 P. R. China; 2 Faculty of Environment and Life, Beijing University of Technology, Beijing, 100124 P. R. China

**Keywords:** Genetics research, Medical genetics

**Dear Editor**,

The SARS-CoV-2 Omicron variant has rapidly displaced the Delta variant and spread across the world. The Omicron variant harbors over 60 mutations, and 15 of the mutations are located in the receptor-binding domain (RBD).^1^ Compared with parental virus and previous variants, the Omicron variant is characterized by decreased hospitalization rates and less severe disease in patients.^2^ However, how the Omicron variant reduces pathogenicity remains unclear. The Omicron variant has diminished fusogenicity, although the underlying mechanism is unknown. Fusogenicity was shown to be associated with pathogenicity in SARS-CoV-2 patients.^3^ The L452R mutation, one of the most frequent mutations (Fig. 1a, b), is the only RBD domain mutation that emerges in the Delta variant but is absent in the Omicron variant (Fig. 1c). It has been reported that L452R mutation increases SARS-CoV-2 fusogenicity and infectivity.^4^ Here, we developed an L452R mutated Omicron variant (Omicron-L452R) and found that the Omicron-L452R variant rescued fusogenicity and strengthened the high infectivity by enhancing the cleavage of the spike protein. Notably, Omicron-L452R greatly enhanced the ability of Omicron to infect lung tissues of humanized ACE2 mice. Furthermore, the Omicron-L452R variant dramatically enhanced glycolysis in host cells. Our data suggest that the decreased fusogenicity of the Omicron variant is due to a lack of the L452R mutation present in the Delta variant.

**Fig. 1:**
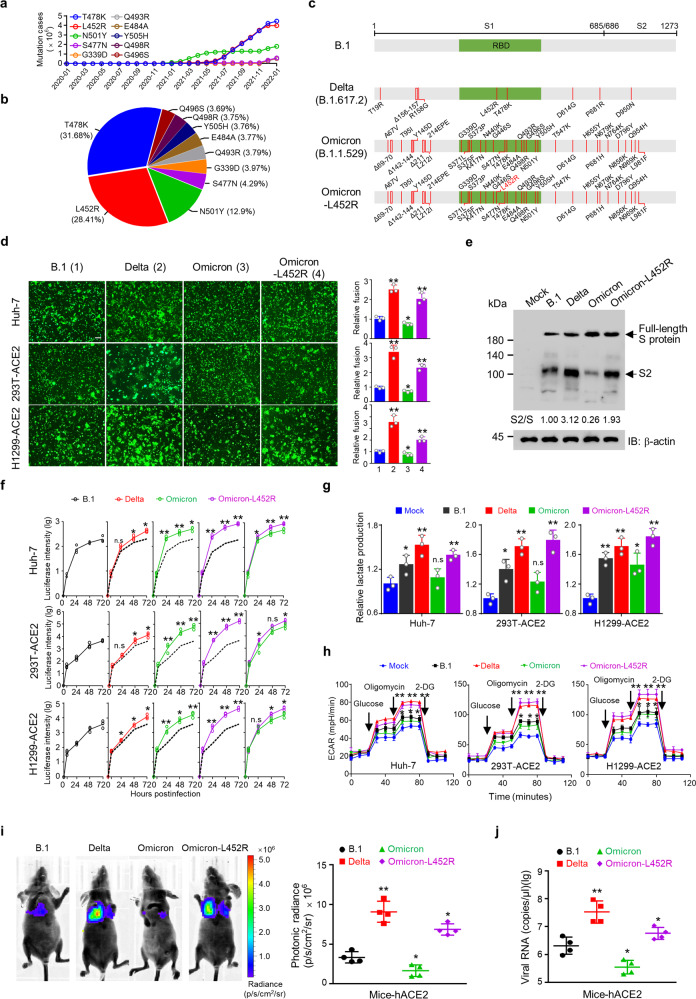
The Omicron-L452R variant increases fusogenicity, infectivity and host glycolysis. **a** The cumulative cases of SARS-CoV-2 virus mutations based on data deposited in the GISAID database from January 2020 to January 2022. **b** Global distribution of SARS-CoV-2 virus mutations as of January 2022. **c** Schematic diagram of the spike mutations of the parental (B.1), Delta, Omicron, and Omicron-L452R variants. **d** Representative images of syncytium formation with the indicated GFP-spike proteins in Huh-7 and hACE2 stably transfected 293T and H1299 cells. The graphs in the right panel show the relative fusion. The results shown are the mean ± SD of three independent experiments. Scale bar, 200 μm. **e** Western blot analysis of SARS-CoV-2 spike cleavage of the indicated variant pseudoviruses in 293T cells stably transfected with hACE2. β-actin was used as a loading control. The densitometry of cleavage was analyzed with Image J. The results shown are the mean ± SD of three independent experiments. **f** Pseudovirus entry of B.1, Delta, the Omicron-L452R variants in Huh-7 cells and 293T and H1299 cells stably transfected with ACE2. Pseudovirus entry was quantified by measuring the luciferase activity in the cell lysates at the indicated times after infection. Fold changes in the luciferase signal were normalized to uninfected cells. The results shown are the mean ± SD of three independent experiments. *P < 0.05, **P < 0.01. **g** Lactate production assay in Huh-7 cells, ACE2 stably transfected 293T and H1299 cells infected with B.1, Delta, the Omicron-L452R variant pseudoviruses. The results shown are the mean ± SD of three independent experiments. *P < 0.05, **P < 0.01. **h** Extracellular acidification rate (ECAR) analysis of glycolysis (following glucose injection), glycolytic capacity (following oligomycin injection), and glycolytic reserve (glycolytic capacity; glycolysis) in Huh-7 cells and ACE2 stably transfected 293T and H1299 cells infected with B.1, Delta, Omicron and the Omicron-L452R variant pseudoviruses. The results shown are the mean ± SD of three independent experiments. *P < 0.05, **P < 0.01. **i** Representative bioluminescence image at 2 days of K18-hACE2 transgenic mice injected with the indicated pseudovirus through the tail vein (2 × 10^6^ TCID50 per mouse). The luminescence signal is represented by an overlaid false-color image with the signal intensity indicated by the scale (right panel) (*n* = 4). **j** qRT–PCR analysis of virus copies in infected mouse lung tissues from (**i**). The results shown are the mean ± SD of three independent experiments. *P < 0.05, **P < 0.01

SARS-CoV-2-infected cells fuse with neighboring cells to form multinucleated cells named syncytia. Syncytia may contribute to virus transmission, immune evasion, and pathogenicity. It has been reported that infected syncytial pneumocytes are present in 87% of deceased SARS-CoV-2 patients, and syncytia formation correlates with disease severity. The Omicron variant has exhibited diminished fusogenicity and pathogenicity compared with the Delta variant and parental virus. Because of the essential role of the L452R mutation in SARS-CoV-2 fusogenicity and infectivity, we investigated the effect of L452R on the fusogenicity of the Omicron variant. Consistent with a previous report, compared to the parental virus (B.1), the Omicron variant almost lost fusogenicity, and the Delta variant demonstrated greatly enhanced fusogenicity. Importantly, the Omicron-L452R variant exerted markedly enhanced fusogenicity (Fig. 1d). Efficient syncytia formation depends on the cleavage of the spike (S) protein at the S1/S2 site.^5^ Next, we investigated the effect of variants and parental virus on spike protein cleavage. In agreement with the syncytia formation assay, the Omicron variant reduced the cleavage of the SARS-CoV-2 S protein compared with the Delta variant and parental virus, whereas the Omicron-L452R variant greatly enhanced the cleavage of the S protein (Fig. 1e). It has been reported that mutations in the Omicron spike protein introduce electrostatic contacts and enhance the interaction between the S1 and S2 subunits, which results in reduced S protein cleavage. Whether the L452R-Omicron variant promotes cleavage of the S protein by decreasing the interaction of the S1 and S2 subunits remains to be investigated.

The Omicron variant spike protein binds human ACE2 and facilitates its entrance and confers Omicron increased infectivity in human primary nasal epithelial cultures and other ACE2-positive cells. Consistent with previous data, the pseudovirus infection assay indicated that the Omicron variant demonstrated enhanced entry into Huh-7, 293T-ACE2, and H1299-ACE2 cells compared to the Delta variant and parental virus, and the Omicron-L452R variant further increased viral entry into these cells (Fig. 1f). In summary, the Omicron-L452R variant not only rescued fusogenicity but also strengthened infectivity, suggesting that the Omicron-L452R variant is a very risky variant.

To obtain available energy and nucleotides for viral replication, SARS-CoV-2 induces metabolic reprogramming in host cells in a manner similar to the Warburg effect in cancer cells. However, the effect of the Delta and Omicron variants on glycolysis has not been investigated. We found that the Delta and Omicron-L452R variants increased lactate production more than the Omicron variant in Huh-7, 293T-ACE2, and H1299-ACE2 cells, although the Omicron variant enhanced lactate production in H1299-ACE2 cells (Fig. 1g). In agreement with lactate production, the extracellular acidification rate (ECAR) assay indicated that the Delta and Omicron-L452R variants highly stimulated glycolysis in Huh7, H1299-ACE2, and 293T-ACE2 cells, while the Omicron and parental viruses slightly increased glycolysis flux (Fig. 1h). Therefore, the Delta and Omicron-L452R variants may use enhanced glycolysis to support their high fusogenicity and high infectivity. It has been shown that SARS-CoV-2 infection increases the expression of hexokinase (HK2) and pyruvate kinase isozyme (PKM), key rate-limiting enzymes in glycolysis, and promotes lactate production, resulting in enhanced host glycolysis. Whether the Omicron-L452R variant stimulates host glycolysis by regulating glycolysis-related enzymes remains to be elucidated.

Next, the infectivity of the Omicron-L452R variant was investigated in K18-hACE2 transgenic mice in vivo. The bioluminescence of infected mice was measured to indicate the pseudovirus infection severity. Consistent with live SARS-CoV-2 virus, the Delta variant has the highest infectivity. The Omicron variant modestly inhibited infectivity, whereas Omicron-L452R significantly enhanced infectivity (Fig. 1i). qRT-PCR was performed to detect the infected pseudovirus copies. Compared to mice infected with the parental virus, the virus copies in the Omicron-infected mouse lung tissues were reduced, while the virus copies in the Omicron-L452R variant-infected mouse lung tissues were markedly enhanced (Fig. 1j). Therefore, the Omicron-L452R variant enhanced the ability of Omicron to infect lung tissues in vivo.

The Omicron variant is characterized as less pathogenic and more transmissible than the parental and previous variants, which may be due to crystal structure changes caused by over 30 mutations in the Spike protein. In this investigation, we found that the further mutation of L452R in the Omicron variant not only rescued fusogenicity but also strengthened the high infectivity. Importantly, the Omicron-L452R variant enhanced the ability of Omicron to infect lung tissues of humanized mice. By dramatically enhancing the anaerobic glycolysis of host cells, the Omicron-L452R variant obtains enough energy and nucleotide material to replicate in human cells. Taken together, the L452R mutation dramatically increases the risk of the Omicron variant. Our data explain why the Omicron variant has decreased fusogenicity and suggest that whether the L452R mutation is present in the Omicron variant should be closely monitored, and specific therapeutic antibodies and vaccines that target the L452R mutation should be developed.

## Supplementary information

Supplementary Materials

## Data Availability

The data used to support the findings of this study are available from the corresponding author upon reasonable request.
