# Supplementary material for: SARS-CoV-2 spike L452R mutation increases Omicron variant fusogenicity and infectivity as well as host glycolysis
Source: Signal Transduct Target Ther. 2022 Mar 9;7:76. doi: 10.1038/s41392-022-00941-z (PMC8905570; doi:10.1038/s41392-022-00941-z)
Supplement: Supplementary file 1 — Supplementary Materials [file 41392_2022_941_MOESM1_ESM.docx]

Supplementary Materials for

SARS-CoV-2 spike L452R mutation increases Omicron variant fusogenicity and infectivity as well as host glycolysis

Yanan Zhang, Ting Zhang, Yihui Fang, Jie Liu, Qinong Ye and Lihua Ding

Correspondence to: Lihua Ding (dinglh2004@126.com) or Qinong Ye (yeqn66@yahoo.com)

**This PDF file includes:**

Materials and Methods

Materials and Methods

**Cell lines and cell culture**

Human embryonic kidney cell line 293T was purchased from American Type Culture Collection (ATCC). Huh-7, 293T-ACE2 and H1299-ACE2 were kindly provided by Xinlong Yan, Junjie Xu and Long Cheng, respectively. All cells were tested for mycoplasma contamination and grown in Dulbecco’s modified eagle’s medium (DMEM, Gibco, USA) with 10% fetal bovine serum (FBS, TIANHANG, China) and 1% Penicillin-Streptomycin Solution 100× (BioMed, China), 37℃ and 5% CO_2_ in humidified air.

**Construction of the pseudoviruses**

Plasmids expressing SARS-CoV-2 S parental (pV-S) or mutant (pV-S-Delta and pV-S-Omicron) protein and their pseudoviruses were obtained from Vazyme Biotech. L452R mutated Omicron variant (pV-S-Omicron-L452R) was constructed based on pV-S-Omicron using Mut Express II Fast Mutagenesis Kit V2 (Vazyme, China), according to the manufacturer’s instructions. To prepare pseudoviruses, 293T cells were cotransfected with psPAX2, CD511B-Fluc and plasmids expressing parental S or its derivatives using Lipofectamine 3000 reagent (Thermo Fisher Scientific, USA). At 48 hours post transfection, the culture supernatants were harvested, centrifuged at 1000×g for 10 min, filtered with 0.45-μm filter, titrated with TCID_50_, aliquoted, and frozen at -80℃ for further use.

**Real-time RT-PCR**

The pseudovirus or total RNA was used as the template for real-time RT-PCR using One Step TB Green PrimeScript PLUS RT-PCR kit (Takara, Japan), according to the manufacturer’s instructions. The primers were as follows: lenti-LTR-F: 5'-TGTGTGCCCGTCTGTTGTGT-3'; lenti-LTR-R: 5'-GAGTCCTGCGTCGAGAGAGC-3'. The fluorescent signal was acquired using a CFX Connect Real-Time PCR Detection system (Bio-Rad, USA).

**Pseudovirus entry assay**

For analysis of S protein-driven cell entry, target cells expressing ACE2 were seeded in 96-well plates and infected by indicated pseudoviruses. The infected cells were harvested at indicated time points and used for detection of luciferase intensity with the One-Lite Luciferase Assay System (Vazyme, China), according to the manufacturer’s instructions.

**Syncytium formation assay**

For preparing effector cells expressing S protein of SARS-CoV-2, plasmids expressing parental S or its derivatives were transfected into 293T cells. Huh-7, 293T-ACE2, and H1299-ACE2 cells, constantly expressing human ACE2 receptors on the membrane surface, were used as target cells. On the next day, the effector cells were collected and resuspended, and were added into target cells in a ratio of 1:1 in a 24-well plate. Then, the effector cells and target cells were cocultured in DMEM containing 10% FBS for 24 h. After incubation, five fields were randomly selected and photographed using a fluorescence microscope. The area of fused cells was calculated by Image J software.

**Extracellular acidification rate assay**

The extracellular acidification rate (ECAR) was measured using the Seahorse XF Glycolysis Stress Test Kit (Agilent Technologies) and the Seahorse XF^e^ 96 Extracellular Flux Analyzer (Seahorse Bioscience). Experiments were performed according to the manufacturer’s instructions. Briefly, cells expressing ACE2 were infected with indicated pseudoviruses. The infected cells were harvested and the cell number was counted. Ten thousand cells per well were then seeded into a Seahorse XF 96 cell culture microplate for 10 h, at which time cell number for each group was very similar. The cells were used for measurement of ECAR. After baseline measurements, glucose, the oxidative phosphorylation inhibitor oligomycin, and the glycolytic inhibitor 2-DG were sequentially injected into each well at the indicated time points. Data were analysed by Seahorse XF-96 Wave software. ECAR was reported in mpH/minute. The results were normalized to cell number.

**Lactate production assay**

For measurement of lactate production, target cells expressing ACE2 were seeded into 24-well plates and infected by the indicated pseudoviruses. After infection for 48 h, cells were harvested for measurement of lactate production using the LA Assay Kit (Biovision, Switzerland) according to the manufacturer’s instructions. The lactate level was measured at 570 nm in a microplate reader and normalized with cell number.

**Western blot assay**

Cells lysates were harvested at 48 h post infection. The protein concentration was determined using a Micro BCA Protein Assay Kit (Thermo Fisher Scientific, USA). Equal amounts of protein were separated by SDS-PAGE, transferred onto the nitrocellulose membrane, incubated in 5% skimmed milk to block non-specific reaction. Specific primary antibodies were incubated with the blocked membranes at 4°C overnight, followed by horseradish peroxidase (HRP) conjugated secondary antibodies for 1 h at room temperature. Full-length S protein and S2 were detected with a rabbit anti-SARS-CoV-2 spike S2 antibody (Sino Biological, China). The signal was developed by Super Signal West Pico PLUS Chemiluminescent Substrate (Thermo Scientific, USA), and detected using ChemiDoc Imaging System (Bio-Rad, USA).

***In vivo* virus challenge in mice**

Animal studies were approved by the Institutional Animal Care Committee of Beijing Institute of Biotechnology. The study used 6-8-week-old K18 human ACE2 transgenic mice, a mouse model expressing human ACE2 (hACE2). The mice were injected with indicated pseudoviruses through tail vein injection (2 × 10^6^ TCID_50_ per mouse). After 2 days, the mice were imaged using the IVIS200 imaging system (Xenogen Corporation, Alameda, CA, USA). After euthanasia, all the infected mice lungs were excised to extract total RNA for analysis of virus copies as described in Real-time RT-PCR.

**Statistical analysis**

Data analyses were performed with GraphPad Prism 7.04. Comparison between two experiment groups was determined by two-tailed Student’s test. Comparison among three or more experiment groups was determined by ANOVA with Tukey’s multiple comparisons by Bonferroni correction. In all assays, p < 0.05 was considered statistically significant.
